# Psychological detachment in Chinese higher education: a multitheoretical model of academic stress, cultural pressure, and coping resources

**DOI:** 10.3389/fpsyg.2025.1647184

**Published:** 2025-10-14

**Authors:** Yingyan Chen, Lily Pan, Feifei Lu, Daokai Sun, Chuanjing Liao, Meng Na

**Affiliations:** ^1^Information Engineering College, Jinhua University of Vocational Technology, Jinhua, Zhejiang, China; ^2^College of Mathematics and Physics, Wenzhou University, Wenzhou, Zhejiang, China; ^3^Pharmaceutical Engineering College, Jinhua University of Vocational Technology, Jinhua, Zhejiang, China; ^4^Center of Mental Health Education, Wenzhou University, Wenzhou, Zhejiang, China; ^5^School of Education, Wenzhou University, Wenzhou, Zhejiang, China; ^6^Graduate School of Business, Universiti Kebangsaan Malaysia, Bangi, Selangor, Malaysia

**Keywords:** psychological detachment, academic stress, digital burnout, filial piety stress, social comparison anxiety

## Abstract

Psychological detachment—the ability to mentally disengage from academic demands during non-study time—remains elusive for Chinese university students, intensified by cultural norms that equate disengagement with weakness and by institutional systems reinforcing constant connectivity. This study integrates the Stressor–Strain–Outcome Model, Conservation of Resources Theory, and Cognitive Appraisal Theory to examine how academic perfectionism, social comparison anxiety, digital burnout, and filial piety stress affect detachment. The mediating roles of resilience, self-esteem, and perceived social support, and the moderating effect of help-seeking stigma, were also tested. A time-lagged survey of 383 students in Zhejiang, analyzed with PLS-SEM, revealed that social comparison anxiety strongly undermines detachment, while digital burnout exerts weaker direct effects but interacts with coping resources. Resilience and self-esteem consistently mediated stressor–detachment links, though stigma diminished their protective effects. These results extend stress-coping theory by showing how cultural obligations and digital immersion constrain recovery in collectivist contexts. The findings highlight the need for resilience training, stigma-reduction initiatives, and digital wellbeing programs. Beyond China, the study underscores how universal stressors such as perfectionism and comparison interact with cultural scripts, offering insights transferable to global higher education.

## 1 Introduction

The intensifying demands of higher education have precipitated a global mental health crisis among university students. Recent meta-analyses indicate that approximately 18.2% exhibit clinically significant symptoms of anxiety and 21.4% show signs of depression (Terlizzi and Zablotsky, 2024). In China, this crisis is particularly acute: nearly 68% of students report chronic academic stress, and more than 21% experience severe emotional exhaustion and cognitive burnout (Sun et al., 2024). These conditions are not merely psychological anomalies but are structurally embedded within a competitive educational system that equates academic excellence with moral virtue, social mobility, and filial duty. Unlike Western systems that emphasize personal growth and psychological balance, China’s higher education landscape—shaped by Confucian norms and performance-driven institutions—creates a rigid meritocracy where underperformance is interpreted not as personal struggle, but as a moral failing that dishonors one’s family (Han and Cheung, 2024). Students are expected to sustain excellence without displaying fatigue, detachment, or emotional disruption (Olson et al., 2025).

This environment generates a student population that is cognitively overloaded, emotionally fatigued, and increasingly incapable of psychological recovery. Within such a context, psychological detachment—the ability to mentally disengage from academic stressors during non-study periods—is not a peripheral concern but a vital mechanism for preserving emotional regulation, cognitive functioning, and long-term wellbeing. Yet detachment remains especially difficult to attain in Chinese higher education due to interwoven academic, institutional, and cultural pressures. Confucian values emphasizing discipline, obedience, and academic loyalty reinforce a moralized view of education in which rest is treated with suspicion and disengagement is equated with irresponsibility (Wu and Chen, 2020). The influence of the *gaokao* system—China’s high-stakes university entrance examination—persists long after admission, fostering a hypercompetitive culture in which 58% of students report filial piety stress tied to parental expectations concerning grades, career choices, and future outcomes (Cheng and Hamid, 2025). Coupled with institutional stressors such as competitive grading, rigid curricula, and limited psychological support, this pressure renders detachment not only rare but socially discouraged (Chung et al., 2025; Zheng et al., 2025).

The digitalization of academic life further exacerbates these challenges. Since the COVID-19 pandemic, remote and hybrid learning have normalized an “always-on” academic culture that blurs boundaries between work and recovery. More than 72% of Chinese university students report excessive screen time due to academic demands, while 64% display symptoms of digital burnout such as sleep disruption, attentional fatigue, and emotional depletion (Jo and Lee, 2021; Wang et al., 2021). Incessant exposure to academic platforms and notifications impairs cognitive rest. At the same time, the widespread use of social media platforms such as WeChat, QQ, and Xiaohongshu intensifies social comparison anxiety: over 90% of students report comparing their academic and career progress with peers online (Ojha et al., 2021), a practice linked to perfectionism, self-doubt, and chronic stress (Mäntymäki et al., 2022). These dynamics create a feedback loop in which students are psychologically tethered to external validation and unable to disengage even during designated rest periods (Flett et al., 2024; Bazine et al., 2025; Piko et al., 2025).

While student mental health challenges are increasingly recognized as a global concern, the Chinese context presents distinctive obstacles. In Western higher education, boundaries between study and recovery are often reinforced by institutional structures and cultural norms emphasizing personal balance, whereas in collectivist East Asian systems, moralized expectations and familial obligations intensify the costs of disengagement. Similar pressures are also observed in other high-performance contexts, such as South Korea and Japan, though with different institutional configurations.

Despite accumulating empirical evidence, current research still fails to capture the multidimensional and culturally embedded determinants of psychological detachment in Chinese higher education. Much of the detachment literature remains dominated by Western occupational models that assume individual autonomy, institutional support, and clear boundaries between work and leisure (Sonnentag and Niessen, 2020). Such assumptions are incompatible with the collectivist logic of China’s academic system, where engagement is reinforced by institutional expectations and moral imperatives. Consequently, existing frameworks lack explanatory power in environments where students are expected to remain perpetually engaged, even at the expense of psychological depletion (Barati-Ahmadabadi et al., 2025; Bravo-Adasme et al., 2025; Wu et al., 2025).

At the construct level, academic stressors such as perfectionism, social comparison anxiety, and digital burnout are often studied in isolation, with limited attention to their cumulative and interactive effects. Their convergence within a high-stakes, digitally mediated, and culturally moralized academic culture has yet to be systematically theorized. Moreover, filial piety stress—a culturally specific form of internalized obligation—remains largely absent from cognitive recovery models, despite its documented role in over-engagement and emotional exhaustion (Wu and Chen, 2020; Olson et al., 2025). This gap underscores a critical limitation: mainstream detachment models rarely consider cultural scripts that discourage disengagement and valorize overwork (Goldstein-Gidoni, 2025; Sheng et al., 2025).

Although resilience, self-esteem, and perceived social support are widely recognized as psychological buffers (Hobfoll, 2010), their mediating roles in enabling detachment under simultaneous academic, technological, and familial pressures remain underexplored. In addition, help-seeking stigma—a well-documented sociocultural barrier in East Asia—moderates whether students access or activate these resources. While empirical studies have linked stigma to reduced service use (Hu et al., 2024), it has not yet been modeled as a barrier to detachment, nor tested as a moderator in resource-based coping frameworks (Wong et al., 2022; Park et al., 2025).

These theoretical and empirical gaps constrain current understandings of why psychological detachment is so elusive in high-performance academic environments, especially those shaped by collectivist and moralized educational cultures. To address this, the present study develops an integrated conceptual framework that synthesizes the Stressor-Strain-Outcome (SSO) Model, Conservation of Resources (COR) Theory, and Cognitive Appraisal Theory (CAT). Each of these frameworks has individually enriched the stress and coping literature, yet their joint application to culturally embedded academic detachment remains conceptually underdeveloped. The SSO model (Sonnentag and Fritz, 2015), though foundational in occupational health psychology, assumes individual agency and structurally bounded work-rest cycles—assumptions that do not align with the normative expectations of continuous academic engagement in Chinese higher education (Tang and Gao, 2025). COR Theory (Hobfoll, 2010), with its emphasis on resource preservation, has explained resilience and burnout but insufficiently addresses how culturally internalized pressures—such as filial piety stress—erode resource reserves (Xu et al., 2025). Similarly, CAT (Lazarus and Folkman, 1984) provides valuable insights into stress appraisal but has yet to be extended to contexts where disengagement is filtered through stigmatized cultural scripts around help-seeking, coping legitimacy, and emotional regulation (Hadad, 2025; Younis, 2025).

Building on this integrated perspective, the present study aims to:

- Examine the effects of academic perfectionism, social comparison anxiety, digital burnout, and filial piety stress on psychological detachment;
- Investigate the mediating roles of resilience, self-esteem, and perceived social support in buffering detachment difficulties;
- Evaluate the moderating effect of help-seeking stigma on the relationship between psychological resources and detachment.

By empirically modeling the intersection of cultural norms, academic expectations, and psychological mechanisms, this study makes a contextually grounded contribution to mental health scholarship. Its findings aim to provide theoretical advancement and practical insights for educators, institutional leaders, and mental health professionals in designing culturally responsive interventions that foster detachment, resilience, and sustainable academic engagement in Chinese higher education (Pevec-Zimmer et al., 2024; Stein et al., 2025).

## 2 Literature review

### 2.1 Psychological detachment

Psychological detachment—mentally disengaging from academic or work-related stressors—is vital for cognitive recovery, wellbeing, and sustained productivity (Sonnentag and Fritz, 2015). While primarily examined in workplace settings, its relevance in academic contexts has grown, particularly among students in high-pressure environments. Rooted in recovery theory, detachment aids psychological restoration by preventing the prolonged cognitive engagement that depletes mental resources, heightening stress and exhaustion (Meijman and Mulder, 1998). However, detachment depends on various psychological, social, and environmental factors.

Perfectionism, social comparison, and digital over-engagement are notable barriers to detachment (Sonnentag and Kühnel, 2016). Students with perfectionistic tendencies struggle to disengage due to rumination and fear of underperformance (Fleming and Dorsch, 2024; McKay et al., 2024; Gonzalez-Hernandez et al., 2025). In collectivist cultures, these tendencies intensify as academic success is closely tied to familial expectations, further exacerbating stress (Liu et al., 2024). Social comparison anxiety compounds detachment problems in the digital age, where continuous exposure to peer achievements fosters self-doubt (Verduyn et al., 2020). Digital burnout presents an additional challenge: constant connectivity and academic notifications blur study-rest boundaries, fostering an “always-on” culture that undermines detachment (Wang et al., 2021). Excessive screen time correlates with poor sleep, cognitive fatigue, and psychological strain (Kühnel et al., 2020). Conversely, deliberate digital disengagement—such as technology detox—promotes detachment and recovery, though few studies differentiate between active (e.g., studying) and passive (e.g., scrolling) digital use (Ahmed et al., 2025).

Nevertheless, psychological and social resources facilitate detachment. Resilience, self-esteem, and social support buffer chronic stress and detachment failure (Hobfoll, 2010). High-resilience individuals exhibit greater cognitive flexibility, enabling them to refocus attention away from stressors (Chen et al., 2022). Self-esteem also plays a critical role: students with low self-worth internalize failures, increasing rumination and stress (Orth and Robins, 2022). Meanwhile, social support from family, peers, and institutions alleviates psychological strain by fostering adaptive coping (Tomás et al., 2024). Students with strong networks report lower stress and greater detachment (Pascoe et al., 2020), although collectivist norms can complicate disengagement when familial expectations demand constant academic effort.

Effective interventions include mindfulness training, structured leisure activities, and behavioral self-regulation. Mindfulness reduces rumination and promotes cognitive disengagement (Querstret et al., 2020), while physical activities, creative hobbies, and immersive experiences restore mental energy (Sonnentag and Kühnel, 2016). However, many studies overlook cultural contexts—particularly in high-achievement environments with strict curricula and parental pressures that limit recovery opportunities. Most existing research also emphasizes workplace detachment, with less attention on how university students manage academic stress (Weigelt et al., 2019), often ignoring institutional support, digital behaviors, and cultural influences.

### 2.2 Filial piety stress

Filial piety, a Confucian value emphasizing respect and obligation toward parents and elders, shapes the psychological and academic experiences of students in collectivist societies (Lin and Wang, 2022). Though generally associated with family harmony, filial piety stress emerges when meeting these expectations becomes emotionally taxing (Wu and Chen, 2020). Academic success is frequently a means of honoring one’s family, leading students in high-pressure environments to feel compelled to excel, which can result in emotional exhaustion and diminished wellbeing (Han and Cheung, 2024).

Filial piety stress manifests in reciprocal and authoritarian forms (Bedford and Yeh, 2021). Reciprocal filial piety—rooted in gratitude and mutual support—correlates with higher life satisfaction, while authoritarian filial piety stresses duty and obedience, often leading to anxiety, depression, and academic pressure (Zhang and Chen, 2022). In Chinese university students, authoritarian filial piety stress has been linked to self-imposed academic perfectionism and fear of failure (Wu and Chen, 2020). This internal pressure can trigger persistent rumination, reduced detachment, and burnout (Huang and Yeh, 2022).

Beyond academic performance, filial piety stress affects mental health and coping. High-stress students may feel caught between personal aspirations and familial obligations, fostering identity conflict (Zhou et al., 2020). This tension appears in career choices, where parental preferences override individual interests, potentially leading to long-term dissatisfaction. Filial obligations can also dissuade help-seeking, as students worry that admitting distress reflects poorly on family honor (Leung and Shek, 2024).

Resilience, self-esteem, and social support mitigate the negative effects of filial piety stress (Dong et al., 2020). Resilient students better manage familial pressures, while higher self-esteem guards against viewing failures as personal inadequacies (Bai et al., 2021). Peers, mentors, and mental health professionals can provide emotional validation, offering students alternative perspectives (Pascoe et al., 2020). Yet, institutional and culturally sensitive mental health supports remain underexamined (Zheng et al., 2020).

Proposed interventions include cognitive reframing, mindfulness training, and structured academic counseling. Reframing encourages students to interpret familial expectations less as burdens, while mindfulness reduces perfectionistic tendencies by promoting emotional regulation (Huang and Yeh, 2022). Counseling that includes family communication strategies helps students align personal goals with familial expectations (Li et al., 2021). However, research rarely addresses the enduring psychological consequences of filial piety stress in areas like career development, identity formation, or help-seeking (Leung and Shek, 2024). Most work concentrates on immediate wellbeing outcomes, neglecting how familial pressures shape long-term mental health and aspirations.

### 2.3 Theoretical underpinning

This study integrates the Stressor-Strain-Outcome (SSO) Model, Conservation of Resources (COR) Theory, and Cognitive Appraisal Theory (CAT) to construct a cohesive framework explaining how Chinese university students experience and cope with psychological detachment difficulties in high-pressure academic environments. Psychological detachment—defined as the ability to mentally disengage from academic demands—is increasingly vital for maintaining emotional balance, cognitive clarity, and academic resilience (Sonnentag and Fritz, 2015). However, in the Chinese higher education context—where achievement is deeply intertwined with familial duty, peer comparison, and digital immersion—students face multiple, simultaneous stressors that challenge this capacity. By systematically integrating these three theories, the study offers a layered understanding of what impedes detachment, why students vary in their coping capacity, and how their personal and cultural interpretations mediate these dynamics.

The SSO Model (Sonnentag and Fritz, 2015) provides the structural process foundation of the framework. It conceptualizes how external demands—referred to here as academic perfectionism (AP), social comparison anxiety (SCA), digital burnout (DB), and filial piety stress (FPS)—generate psychological strain, which subsequently impairs outcomes such as psychological detachment (PD). This processual logic has been widely applied in occupational health research but remains underutilized in academic settings. In applying the SSO model to student populations, this study highlights how the cumulative effect of these multidimensional stressors leads to emotional exhaustion, cognitive overload, and difficulty disengaging from academic preoccupations. Crucially, the inclusion of filial piety stress introduces a culturally specific form of strain, rooted in Confucian ideals, that amplifies the emotional burden of academic life. Thus, SSO defines the “what happens” in the progression from stressor exposure to impaired recovery, offering a foundation upon which deeper psychological mechanisms can be explored (Rauch et al., 2018; Guo et al., 2025).

While the SSO model outlines this linear progression, it does not explain why some students are better able to cope than others when exposed to the same stressors. Here, Conservation of Resources (COR) Theory (Hobfoll, 2010) enriches the framework by introducing a motivational explanation centered on psychological resources. COR theory posits that individuals seek to conserve and protect valued resources—such as resilience (REL), self-esteem (SE), and perceived social support (PSS)—and experience stress when these resources are threatened or depleted. In this study, students who report higher levels of resilience and support are more likely to buffer the negative effects of AP, SCA, DB, and FPS, enabling more successful psychological detachment. However, stressors like digital burnout and filial piety stress function as resource-draining forces—leaving students cognitively exhausted, emotionally taxed, and less capable of disengaging. Digital burnout is particularly critical in the Chinese context, where the “always-on” culture of online platforms blurs academic and leisure boundaries, continuously consuming students’ psychological resources. Unlike temporary strains, burnout represents a chronic depletion spiral that not only drains energy but also prevents resource replenishment, thereby compounding detachment failure (Bhugra, 2025). By extending COR theory to a collectivist educational setting, this study emphasizes that resource loss is not only internal (e.g., loss of confidence or optimism) but also culturally shaped—students may feel morally obligated to persist under pressure, even when emotionally depleted. Thus, COR addresses the “why it matters” dimension—explaining variations in coping effectiveness through the presence or erosion of psychological resources.

However, even the availability of resources does not guarantee their activation or effectiveness—especially in cultures where seeking support is stigmatized or interpreted as weakness. This leads to the third layer of the model: Cognitive Appraisal Theory (CAT) (Lazarus and Folkman, 1984), which offers a subjective interpretation lens. CAT posits that individuals evaluate stressors through primary (is this threatening?) and secondary (can I cope?) appraisals. Students’ ability to detach is thus influenced by how they cognitively interpret academic challenges and their own coping capacity. For instance, those with high self-esteem may appraise academic perfectionism as a motivating challenge, while those with low self-worth may see it as overwhelming, triggering over-engagement and emotional fatigue. Importantly, help-seeking stigma (HSS) plays a crucial moderating role in this appraisal process: students who perceive stigma in asking for help may reinterpret their stress as shameful or socially illegitimate, further inhibiting detachment. In collectivist cultures such as China, stigma is intensified by filial and cultural expectations of endurance, which position help-seeking as dishonorable or as a failure of resilience. This means that stigma not only prevents students from using available resources but also reshapes their cognitive appraisals, leading them to normalize over-engagement and underreport distress (Kleinman et al., 2024; Pasman et al., 2025). Thus, CAT clarifies the “how students make meaning” of their stress—revealing that cognitive framing and cultural beliefs fundamentally shape whether detachment is seen as permissible, achievable, or desirable.

**Figure 1:**
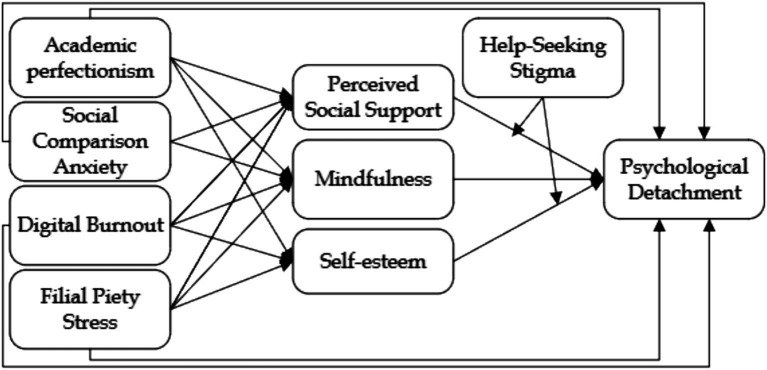
Research framework.

Together, these three theories contribute distinct yet complementary components to the framework (see Figure 1). The SSO Model provides the structural flow, explaining how academic perfectionism, social comparison anxiety, digital burnout, and filial piety stress lead to psychological strain and ultimately impair psychological detachment. COR Theory accounts for individual variability by showing how internal resources—such as resilience, self-esteem, and perceived social support—buffer or exacerbate the effects of stress, depending on their availability and depletion. CAT adds a cognitive layer by illustrating how students’ appraisals of stressors, shaped by self-perceptions and cultural stigma (e.g., help-seeking stigma), influence whether these resources are activated or suppressed. Together, the integration of process (SSO), resource dynamics (COR), and subjective interpretation (CAT) offers a comprehensive understanding of detachment in a culturally and academically intense environment.

By interlinking process, motivation, and perception, this integrated model offers a holistic and culturally grounded explanation for why detachment remains elusive for many students in high-pressure academic environments. The theoretical contribution lies in reconceptualizing psychological detachment not as a simple behavioral outcome, but as a dynamic interplay of external demands, internal resources, and cognitive-cultural meaning-making. In doing so, this study extends each framework beyond its traditional domain—applying SSO to educational stress, expanding COR to include culturally specific depletion (e.g., FPS and chronic digital burnout), and refining CAT to account for stigma-mediated appraisal. The result is a more nuanced and context-sensitive understanding of academic recovery processes in collectivist societies.

### 2.4 Hypothesis development

#### 2.4.1 Academic perfectionism, social comparison, filial piety, and psychological detachment

Academic perfectionism significantly shapes student success and mental wellbeing through two dimensions: perfectionistic strivings and perfectionistic (Fernández-García et al., 2022; Aman and Aman, 2023). Perfectionistic strivings correlate with higher academic achievement, while perfectionistic concerns—defined by excessive self-criticism and fear of negative outcomes—promote anxiety and vulnerability. Conscientious perfectionism fosters wellbeing, whereas self-evaluative perfectionism impairs psychological health (Kamushadze et al., 2021). Given this nuanced dynamic, students with strong perfectionistic concerns may struggle more to psychologically detach from academic tasks, fueling stress and emotional exhaustion.

Social comparison anxiety likewise emerges as a major stressor, undermining self-esteem and mental health (Goodman et al., 2021). Ability-based comparisons often erode psychological capital, whereas opinion-based comparisons can occasionally enhance it (Mao et al., 2020). However, excessive social comparisons, particularly on social media, amplify anxiety, depression, and psychological distress (Ojha et al., 2021). While strategic detachment through social networks offers some relief (Mäntymäki et al., 2022), continuous comparison undermines students’ capacity to disengage from performance-related pressures.

Reliance on digital tools and platforms also contributes to digital burnout, marked by chronic cognitive overload and emotional exhaustion (Huyghebaert-Zouaghi et al., 2022). Mastery of digital competence helps reduce these burdens (Wang et al., 2021). Interventions involving cognitive behavioral therapy (CBT) and mindfulness further support psychological detachment (Tement et al., 2020). Nonetheless, persistent overengagement with digital platforms raises stress and hinders detachment (Jo and Lee, 2021).

Filial piety, particularly in Chinese cultural contexts, significantly affects student wellbeing. Reciprocal filial piety correlates with higher life satisfaction and lower psychological distress, whereas authoritarian filial piety elevates stress (Huang and Yeh, 2022). Mediators such as autonomy, regulatory focus, and attachment style help shape these outcomes (Zhang and Chen, 2022). At work, filial piety can intensify stress–turnover relationships (Li et al., 2021). Students experiencing substantial filial piety stress often find it particularly hard to disengage from academic duties due to strong familial expectations. Based on these insights, we propose:

*H1a*: Academic perfectionism (AP) negatively associates with Psychological Detachment (PD).

*H1b*: Social comparison anxiety (SCA) negatively associates with PD.

*H1c*: Digital burnout (DB) negatively associates with PD.

*H1d*: Filial piety stress (FPS) negatively associates with PD.

#### 2.4.2 Moderated effects: help-seeking stigma

Help-seeking stigma—encompassing both self-stigma and public stigma—can obstruct mental wellbeing by deterring individuals from seeking support (Özdemir et al., 2023). Those with robust social support networks tend to mitigate stigma’s negative impact, whereas individuals with high self-stigma may fail to leverage available support effectively, escalating distress and hampering detachment (Isaac and Buchanan, 2021). Cultural factors, including international student status, further shape help-seeking attitudes (Maeshima and Parent, 2020). Self-esteem also moderates stigma’s psychological effects (Yu et al., 2022), with high self-esteem buffering stigma’s impact and low self-esteem magnifying it (Topkaya, 2021). Adolescents, for instance, might recommend professional help to peers yet hesitate to seek it themselves (Villatoro et al., 2022). Accordingly, we hypothesize:

*H2a:* The interaction between Help-Seeking Stigma (HSS) and Perceived Social Support (PSS) influences PD.

*H2b*: The interaction between HSS and Self-Esteem (SE) influences PD.

#### 2.4.3 Mediated effects: perceived social support

Perceived Social Support (PSS) is pivotal in fostering psychological wellbeing and buffering the detrimental effects of academic stress, perfectionism, and burnout. It mediates the relationship between self-esteem and wellbeing (Poudel et al., 2020) and between academic stress and wellbeing (Poots and Cassidy, 2020). PSS also diminishes the impact of attachment insecurity on loneliness and strengthens resilience (Khan et al., 2024). Among international students, PSS mitigates maladaptive perfectionism’s effects on college adjustment (Lee et al., 2020), lowers distress after disasters (Yuan et al., 2021), mediates the connection between burnout and wellbeing (Rehman et al., 2020), and buffers the link between perceived stress and job burnout (Liu et al., 2022). Therefore:

*H3a*: PSS mediates the negative AP–PD relationship.

*H3b*: PSS mediates the negative DB–PD relationship.

#### 2.4.4 Resilience as a mediator

*Resilience* equips individuals to cope with adversity, adapt to challenges, and sustain mental health, thereby affecting students’ capacity to detach from academic concerns. It buffers academic stress and curbs problematic smartphone use, helping avert psychological distress and burnout (Chen et al., 2022). Resilience also mediates the association between social comparison and wellbeing (Mao et al., 2020; Güler et al., 2024), reducing the negative effects of frequent comparisons. In digital contexts, it counters social media overuse and smartphone dependence, preserving emotional balance (Ahmed et al., 2024). Regarding filial piety stress, resilience can facilitate healthier coping strategies that help balance family demands with psychological wellbeing (Zheng et al., 2020). Hence:

*H4a*: Resilience (REL) mediates the negative AP–PD relationship.

*H4b*: REL mediates the negative SCA–PD relationship.

*H4c*: REL mediates the negative DB–PD relationship.

*H4d*: REL mediates the negative FPS–PD relationship.

#### 2.4.5 Self-esteem as a mediator

Self-esteem serves as another crucial psychological resource, shielding students from negative outcomes linked to academic stress and perfectionism. It fosters resilience and adaptive coping (Awad et al., 2022; Fekih-Romdhane et al., 2023), predicts academic engagement via self-efficacy (Zhao et al., 2021), and moderates the impact of work-related stress on detachment (Huyghebaert-Zouaghi et al., 2022). In cultural settings emphasizing filial piety, self-esteem may be compromised when self-worth depends on meeting familial standards (Bai et al., 2021). Additionally, self-esteem mediates links between loneliness and psychological wellbeing (ÇİÇEK, 2022). Consequently:

*H5a*: Self-Esteem (SE) mediates the negative AP–PD relationship.

*H5b*: SE mediates the negative FPS–PD relationship.

## 3 Research method

### 3.1 Data collection

This study employed a time-lagged, three-wave survey design to examine psychological detachment among university students in Zhejiang Province, China. A purposive sampling strategy was adopted to target undergraduate and postgraduate students who were simultaneously exposed to rigorous academic demands and strong familial expectations, as this population reflects the intersection of academic stress and cultural obligations most relevant to the research objectives.

Eligibility criteria required participants to-

(i) have completed at least 1 year of study at their current institution and
(ii) maintain regular communication with parents or guardians regarding academic performance and career expectations.

Individuals not meeting these criteria were excluded. Consistent with Kautish et al. (2020), participants confirmed eligibility by reporting their academic workload (program and year of study) and family expectations (frequency of parental discussions, perceived pressure).

To maximize diversity and enhance representativeness within Zhejiang’s higher education context, invitations were distributed across multiple academic disciplines using university networks, student associations, and social media platforms. While purposive sampling was used, stratification by gender, age, discipline, and family background (see Table 1) ensured that the final sample reflected a broad cross-section of student experiences. Each participant was assigned a unique identifier to link responses across all three waves.

**Table 1:** Demographics of the respondents.

Variables	Category	*N* (%)
Gender	Male	176 (45.9%)
	Female	200 (52.2%)
	Other/prefer not to say	7 (1.8%)
Age group	18–20	112 (29.2%)
	21–23	165 (43.1%)
	24–26	80 (20.9%)
	27 and above	26 (6.8%)
Academic level	Undergraduate	279 (72.8%)
	Postgraduate	104 (27.2%)
Field of study	STEM	98 (25.6%)
	Social Sciences	85 (22.2%)
	Business & Economics	72 (18.8%)
	Humanities & Arts	88 (23.0%)
	Vocational	33 (8.61%)
	Other	40 (1.82%)
Parental education level	No formal education	12 (3.1%)
	Primary education	34 (8.9%)
	Secondary education	140 (36.6%)
	Higher education	197 (51.4%)
Parental income level (annual)	<50,000 CNY	90 (23.5%)
	50,000–100,000 CNY	140 (36.6%)
	100,000–200,000 CNY	105 (27.4%)
	>200,000 CNY	48 (12.5%)
Frequency of parental academic discussions	Daily	102 (26.6%)
	Weekly	185 (48.3%)
	Monthly	75 (19.6%)
	Rarely	21 (5.5%)
Perceived family pressure (self-reported)	Low	49 (12.8%)
	Moderate	154 (40.2%)
	High	127 (33.2%)
	Very high	53 (13.8%)

The time-lagged design, with data collected at 1-month intervals, was implemented to reduce common method bias (MacKenzie et al., 2011) and to strengthen temporal ordering for causal inference (Mehmood et al., 2023). By separating the measurement of predictor and outcome variables, the design minimized inflated associations and encouraged participants to reflect more accurately on their experiences (Podsakoff et al., 2003).

A pilot study with 25 students, reviewed by two experts in Chinese cultural psychology, confirmed the cultural appropriateness and conceptual alignment of the measures for psychological detachment and filial piety stress. Only minor wording adjustments were necessary for clarity. The research protocol received approval from the institutional Research Ethics Committee, and all participants provided informed consent, with assurances of anonymity, confidentiality, and voluntary participation.

### 3.2 Sample size and statistical power

Prior to full data collection, an *a priori* power analysis was conducted to estimate the minimum required sample size. Following Cohen’s (1992) guidelines, assuming a medium effect size (*f*^2^ = 0.15), statistical power of 0.80, seven predictors, and *α* = 0.05, the minimum requirement was 108 participants (Hair et al., 2022). However, given the study’s multi-wave design and the inclusion of indirect (mediation) and interaction (moderation) effects—both of which typically demand larger samples to achieve stable parameter estimates—a more conservative target of ≥350 participants were established.

To achieve this, 500 invitations were distributed, yielding:

*Wave 1 (T1)*: 451 valid responses (demographics, stressors, baseline wellbeing).

*Wave 2 (T2)*: 410 responses (filial piety stress, resilience, detachment).

*Wave 3 (T3)*: 383 valid responses (academic outcomes, help-seeking behaviors).

The final analytic sample of 383 participants exceeded both the a priori minimum and the conservative target. With this sample, the study retained >0.80 power to detect small-to-moderate path coefficients (|*β*| ≈ 0.10–0.15) in the structural model, while also ensuring adequate sensitivity for indirect effects. Monte Carlo simulations with 5,000 replications confirmed that indirect effects in the. 03–0.08 range achieved ≥0.80 power with bias-corrected bootstrapping, validating the adequacy of the sample for mediation testing.

The overall response rate of 76.6% is high for a three-wave panel survey. Non-response bias was evaluated by comparing early and late respondents, with no significant differences observed across key demographic and academic variables, supporting the representativeness of the dataset.

This staggered, multi-wave design minimized common method variance and captured temporal dynamics of academic and cultural stressors. The resulting dataset—encompassing diverse academic levels, fields of study, and family contexts (see Table 1)—provides a statistically robust and contextually rich foundation for analyzing psychological detachment in Chinese higher education.

### 3.3 Measurement items development

All constructs, variables, and scale items in this study were adapted from well-established prior research to ensure validity and reliability. Academic Perfectionism (AP) was measured using three items adapted from Stoeber and Otto (2006) and Madigan (2019), capturing students’ tendencies toward high academic standards and dissatisfaction with imperfection. Digital Burnout (DB) was assessed with three items based on Reinecke and Eden (2017) and Wang et al. (2021), reflecting emotional exhaustion and cognitive overload due to excessive digital engagement. Filial Piety Stress (FPS) was measured using four items adapted from Bedford and Yeh (2021) and Wu and Chen (2020), focusing on students’ perceived pressure to fulfill familial academic expectations. Help-Seeking Stigma (HSS) was assessed through three items adapted from Vogel (2006) and Mackenzie et al. (2004), evaluating reluctance to seek psychological support due to perceived social and familial consequences. Psychological Detachment (PD) was measured with four items from Sonnentag and Niessen (2020) and Weigelt et al. (2019), assessing difficulties in disengaging from academic tasks during leisure time. Perceived Social Support (PSS) was evaluated using three items from Cheng and Chan (2004), reflecting the extent to which students perceive support from family, peers, and institutions. Resilience (REL) was measured with three items adapted from Connor and Davidson (2003), capturing students’ ability to recover from academic setbacks and maintain emotional stability. Social Comparison Anxiety (SCA) was assessed with three items adapted from Verduyn et al. (2020), examining distress caused by academic-related comparisons with peers. Self-Esteem (SE) was measured with three items adapted from Orth and Robins (2022), reflecting students’ overall confidence in their academic abilities and self-worth. All scale items were assessed on a five-point Likert scale, ranging from 1 = strongly disagree to 5 = strongly agree. The measurement properties and scale items are detailed in Table 2.

**Table 2:** Measurement model statistics.

Construct	Code	Items	OL	VIF	CA	CR	AVE
AP	AP1	I strive for perfection in my academic work.	0.892	2.408	0.855	0.912	0.776
	AP2	I have high standards for my academic performance.	0.905	2.650			
	AP3	I feel dissatisfied with my work if it is not perfect.	0.844	1.790			
DB	DB1	I feel emotionally drained from my digital device usage.	0.882	1.997	0.824	0.894	0.738
	DB2	I feel tired when I have to use digital devices for academic purposes.	0.818	1.741			
	DB3	I feel overwhelmed by the amount of time I spend on digital devices.	0.876	1.888			
FPS	FPS1	I feel pressured to meet my parents’ academic expectations.	0.867	2.322	0.880	0.918	0.736
	FPS2	I worry about disappointing my parents with my academic performance.	0.851	2.205			
	FPS3	I feel stressed due to the obligations I have towards my family.	0.849	2.267			
	FPS4	I feel that my family’s expectations place a burden on me.	0.863	2.306			
HSS	HSS1	I would feel embarrassed if my friends knew I was seeking professional help for my academic problems.	0.905	2.276	0.813	0.889	0.728
	HSS2	I would worry about my family’s reaction if they found out I was seeking academic counseling.	0.857	1.831			
	HSS3	I avoid seeking help for academic-related stress to not appear weak.	0.794	1.655			
PD	PD1	I find it difficult to stop thinking about academic work during my free time.	0.840	2.227	0.895	0.927	0.761
	PD2	Even when I try to relax, thoughts about my studies intrude.	0.881	2.665			
	PD3	I struggle to disconnect from academic responsibilities outside of study hours.	0.915	3.782			
	PD4	I feel guilty when I take a break from my academic tasks.	0.851	2.847			
PSS	PSS1	I can rely on my family for support when I feel stressed.	0.876	2.229	0.853	0.911	0.773
	PSS2	I receive encouragement from friends when facing academic difficulties.	0.904	2.357			
	PSS3	I feel supported by my university in managing academic stress.	0.856	1.877			
REL	REL1	I am able to recover quickly from setbacks.	0.846	1.962	0.831	0.898	0.746
	REL2	I adapt well to changes and challenges in my academic environment.	0.877	1.941			
	REL3	I remain optimistic even when facing significant academic stress.	0.868	1.829			
SCA	SCA1	I frequently compare my academic performance to that of my peers.	0.884	1.994	0.782	0.874	0.698
	SCA2	I feel stressed when I perceive others performing better than me.	0.817	1.549			
	SCA3	I often feel inadequate when I see my classmates achieving success.	0.803	1.620			
SE	SE1	I feel confident in my academic abilities.	0.895	2.445	0.849	0.908	0.768
	SE2	I believe I am just as capable as my peers in my studies.	0.887	2.071			
	SE3	I have a positive self-perception despite academic challenges.	0.846	1.906			

### 3.4 Data analysis strategy

To test the hypothesized relationships, this study employed Partial Least Squares Structural Equation Modeling (PLS-SEM) using SmartPLS 4. PLS-SEM is widely recognized for its ability to handle complex models, small sample sizes, and non-normal data distributions (Hair et al., 2022). Unlike covariance-based SEM, PLS-SEM focuses on maximizing variance explanation in the endogenous constructs, making it suitable for psychological and behavioral studies where predictor variables are (Alam et al., 2025).

A normality test was conducted, and the skewness and kurtosis values of all items fell within the acceptable range of ±1, confirming no significant deviation from normality. Principal Component Analysis (PCA) was performed to verify the unidimensionality of the constructs, applying the Kaiser criterion (i.e., eigenvalues >1). The Kaiser–Meyer–Olkin (KMO) measure yielded a value of 0.84, and Bartlett’s test of sphericity was significant at *p* < 0.001, confirming that the dataset was appropriate for factor analysis (Hair et al., 2022). Additionally, variance inflation factors (VIFs) were all below the threshold of 5, confirming that multicollinearity was not an issue (Hair and Alamer, 2022).

To assess construct validity and reliability, convergent validity was established through composite reliability (CR) values above 0.7 and average variance extracted (AVE) exceeding 0.5 (Fornell and Larcker, 1981). Discriminant validity was verified using the Heterotrait-Monotrait (HTMT) ratio (refer to Table 3) and the Fornell–Larcker criterion (refer to Table 4), ensuring that each construct was distinct from others.

**Table 3:** Heterotrait–Monotrait (HTMT) ratio.

Construct	AP	DB	FPS	HSS	PD	PSS	REL	SCA	SE
AP									
DB	0.743								
FPS	0.803	0.804							
HSS	0.844	0.846	0.801						
PD	0.653	0.701	0.732	0.740					
PSS	0.826	0.756	0.807	0.810	0.635				
REL	0.792	0.750	0.791	0.811	0.708	0.746			
SCA	0.635	0.730	0.827	0.779	0.753	0.707	0.722		
SE	0.806	0.656	0.727	0.820	0.497	0.836	0.776	0.609	

**Table 4:** Fornell–Larcker criterion.

Construct	AP	DB	FPS	HSS	PD	PSS	REL	SCA	SE
AP	0.881								
DB	0.634	0.859							
FPS	0.698	0.753	0.858						
HSS	0.705	0.701	0.751	0.853					
PD	−0.573	−0.602	−0.651	−0.637	0.872				
PSS	0.707	0.645	0.703	0.732	−0.560	0.879			
REL	0.673	0.634	0.687	0.671	−0.621	0.637	0.863		
SCA	0.520	0.588	0.687	0.623	−0.628	0.583	0.589	0.835	
SE	0.690	0.559	0.636	0.719	−0.440	0.773	0.657	0.505	0.876

## 4 Analysis

### 4.1 Measurement model assessment

**Figure 2:**
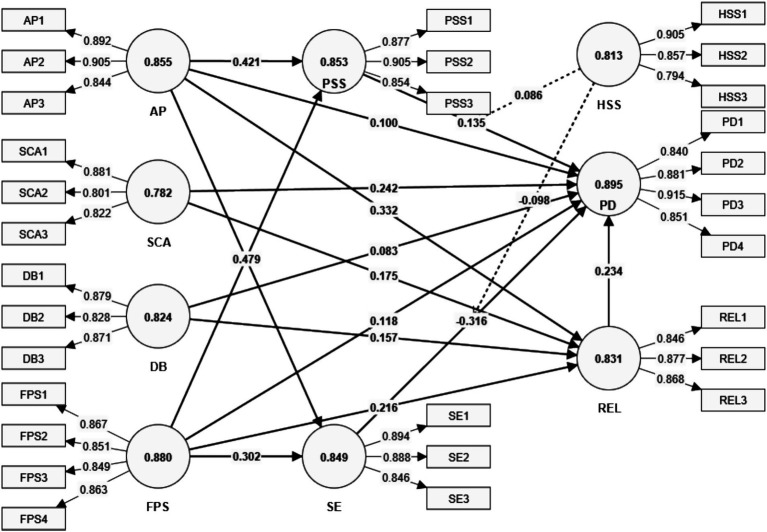
Measurement model.

The measurement model (refer to Figure 2) was evaluated using Partial Least Squares Structural Equation Modeling (PLS-SEM), focusing on reliability, convergent validity, and discriminant validity. Reliability was assessed using Cronbach’s alpha (CA) and composite reliability (CR), both of which exceeded the recommended threshold of 0.70 (Hair et al., 2022). The constructs demonstrated high internal consistency, with CA values ranging from 0.782 to 0.895 and CR values exceeding 0.874, confirming the robustness of the measurement instruments (refer to Table 2).

Convergent validity was established by examining the Average Variance Extracted (AVE), where all constructs exceeded the recommended threshold of 0.50 (Fornell and Larcker, 1981), with AVE values ranging from 0.698 to 0.879. Furthermore, standardized factor loadings for all measurement items were above the minimum acceptable threshold of 0.70, indicating that each item strongly contributed to its corresponding construct.

Discriminant validity was tested using the Fornell–Larcker criterion and the Heterotrait-Monotrait (HTMT) ratio. The square root of each construct’s AVE was greater than its correlations with other constructs, ensuring distinctiveness between variables. Additionally, all HTMT ratios were below the conservative threshold of 0.85 (Henseler et al., 2014), further supporting discriminant validity.

Multicollinearity was assessed through Variance Inflation Factor (VIF) values, which remained well below the critical threshold of 5 (Hair et al., 2022), with the highest being 3.782 for PD3, indicating no severe collinearity concerns among the predictor variables. This ensures that the constructs operate independently without statistical redundancy.

The final assessment of the measurement model confirms its robustness, demonstrating strong reliability, convergent validity, and discriminant validity, with no substantial multicollinearity concerns.

### 4.2 Predictive relevance and model fit statistics

The predictive relevance and model fit statistics (refer Table 5) were assessed using *R*^2^, adjusted *R*^2^, *Q*^2^ predict, RMSE, and MAE, providing insights into the explanatory power and predictive accuracy of the model. *R*^2^ values indicate the proportion of variance explained by the predictor variables, with PSS (0.607), REL (0.575), PD (0.573), and SE (0.528) demonstrating moderate to strong explanatory power, suggesting that the independent variables significantly contribute to explaining these constructs (Hair et al., 2017, 2022). The adjusted *R*^2^ values, which account for model complexity and prevent overfitting, were slightly lower but remained consistent with *R*^2^, confirming the robustness of the model and reducing the risk of model overestimation (Hair et al., 2022).

**Table 5:** Model fit and predictive statistics.

Construct	*R* ^2^	*R*^2^ adjusted	*Q*^2^ predict	RMSE	MAE
PD	0.573	0.561	0.509	0.705	0.529
PSS	0.607	0.603	0.588	0.647	0.456
REL	0.575	0.570	0.558	0.670	0.495
SE	0.528	0.523	0.511	0.704	0.518

The *Q*^2^ predict values, obtained through cross-validated redundancy measures, assess the predictive relevance of the constructs. A *Q*^2^ predict value above zero indicates that the model exhibits substantial predictive relevance (Fornell and Larcker, 1981). The results confirm that PSS (0.588), REL (0.558), PD (0.509), and SE (0.511) all exhibit strong predictive ability, reinforcing the model’s capability in accurately forecasting outcomes. According to Shmueli et al. (2019), a high *Q*^2^ predict value suggests that the model’s endogenous constructs are well predicted by the independent variables, which supports its external validity.

Further evaluation using Root Mean Square Error (RMSE) and Mean Absolute Error (MAE) provided additional validation of the model’s predictive accuracy. RMSE captures the standard deviation of prediction errors, while MAE measures the average absolute differences between predicted and actual values, both of which help in assessing model precision (Hair and Alamer, 2022). PSS recorded the lowest RMSE (0.647) and MAE (0.456), followed by REL (RMSE = 0.670, MAE = 0.495), PD (RMSE = 0.705, MAE = 0.529), and SE (RMSE = 0.704, MAE = 0.518). Lower RMSE and MAE values indicate smaller prediction errors, confirming the model’s predictive strength and robustness (Sarstedt et al., 2020).

Taken together, these results confirm that the model exhibits strong explanatory power and predictive relevance, with all constructs demonstrating significant variance explanation and prediction accuracy. The findings establish a well-fitted and reliable model, ensuring confidence in the subsequent structural path analysis and hypothesis testing (Henseler et al., 2014). Given that both *Q*^2^ predict values and RMSE/MAE results suggest strong model performance, these findings reinforce that the model offers reliable and valid insights into the relationships among psychological detachment, stressors, and coping mechanisms.

### 4.3 Hypothesis testing and discussion

**Figure 3:**
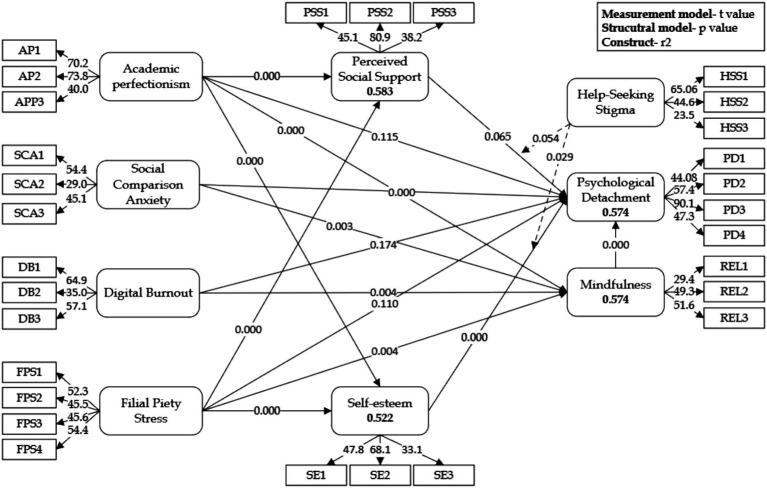
Structural model.

The structural model (Figure 3) was assessed using Partial Least Squares Structural Equation Modeling (PLS-SEM) with bootstrapping (5,000 resamples) to examine the relationships between academic stressors, psychological detachment (PD), and the mediating and moderating influences of psychological resources. Table 6 provides the empirical results, which offer important insights into how academic stress and cultural obligations may be linked to students’ ability to detach in the Chinese higher education context.

**Table 6:** Structural model statistics.

Hypothesis path		Original sample	Standard deviation	*T* statistics	*p_–_*values	*f* ^2^	Support
H1a	AP → PD	−0.100	0.063	1.577	0.115	0.008	No
H1b	SCA → PD	−0.236	0.064	3.709	0.000	0.062	Yes
H1c	DB → PD	−0.081	0.061	1.313	0.189	0.006	No
H1d	FPS → PD	−0.123	0.073	1.682	0.093	0.009	Partially
H2a	HSS × PSS → PD	−0.087	0.044	1.960	0.050	0.010	Yes
H2b	HSS × SE → PD	0.099	0.045	2.205	0.027	0.012	Yes
H3a	AP → PSS → PD	−0.051	0.029	1.769	0.077		Partially
H3b	DB → PSS → PD	−0.020	0.013	1.509	0.131		No
H4a	AP → REL → PD	−0.078	0.026	2.994	0.003		Yes
H4b	SCA → REL → PD	−0.042	0.018	2.319	0.020		Yes
H4c	DB → REL → PD	−0.038	0.015	2.454	0.014		Yes
H4d	FPS → REL → PD	−0.051	0.022	2.300	0.021		Yes
H5a	AP → SE → PD	0.146	0.035	4.152	0.000		Yes
H5b	FPS → SE → PD	0.068	0.028	2.449	0.014		Yes

The analysis revealed mixed evidence regarding the hypothesized associations between academic stressors and PD. Academic perfectionism (AP) did not show a statistically significant association with PD (H1a: *β* = −0.100, *p* = 0.115). While perfectionism has often been conceptualized as a major contributor to academic stress and maladaptive coping (Fernández-García et al., 2022; Aman and Aman, 2023), the present results suggest that perfectionistic tendencies alone do not necessarily translate into lower levels of detachment. This finding resonates with the literature on the multidimensionality of perfectionism, where perfectionistic strivings sometimes correlate with adaptive functioning, while perfectionistic concerns align more strongly with maladaptive outcomes (Stoeber and Otto, 2006). Similar patterns have been documented in Western contexts, where adaptive perfectionism is associated with persistence and achievement but not with burnout (Smith et al., 2023). This suggests that perfectionism’s role in detachment may not be unique to China but varies cross-culturally depending on whether strivings or concerns dominate.

In contrast, social comparison anxiety (SCA) demonstrated a robust and statistically significant negative association with PD (H1b: *β* = −0.236, *p* < 0.001, *f*^2^ = 0.062). This finding indicates that students who frequently compare their academic performance with peers tend to struggle more in psychologically disengaging from academic concerns. The relatively strong effect size highlights SCA as a key factor limiting detachment, consistent with research showing that constant exposure to peers’ achievements—often magnified through social media—fosters rumination, erodes self-worth, and amplifies stress (Goodman et al., 2021). In Europe and North America, similar evidence shows that social media–driven comparison impairs student wellbeing and detachment (Verduyn et al., 2020), although the intensity of effects appears stronger in collectivist contexts such as China and Korea where academic success is closely tied to family honor (Park et al., 2025). These cross-national findings suggest that while SCA undermines detachment globally, cultural norms determine the magnitude of its influence.

Digital burnout (DB) did not show a significant association with PD (H1c: *β* = −0.081, *p* = 0.189). While prior studies in Europe and the U.S. consistently link digital overuse to cognitive fatigue and poor recovery (Kühnel et al., 2020), our results imply that Chinese students may adopt compensatory strategies—such as alternating between study-related and leisure digital use—that buffer against direct detachment failures. In contrast, research in Japan and South Korea reports stronger direct effects of digital exhaustion on detachment (Shin et al., 2023), highlighting how national digital cultures mediate outcomes. This points to the importance of examining not only the amount of digital use but also the purposes and regulation strategies across cultural contexts.

Filial piety stress (FPS) exhibited a marginally significant negative association with PD (H1d: *β* = −0.123, *p* = 0.093). Although weaker than anticipated, this suggests that familial obligations and parental expectations complicate detachment. While this construct is culturally specific to East Asia, parallels exist in Mediterranean and South Asian contexts, where family honor and intergenerational expectations similarly shape students’ academic engagement and stress. Compared to Western settings, where parental pressure is often framed as individual motivation rather than moral duty, the Confucian-rooted dimension of FPS provides a culturally distinctive pathway to detachment difficulties.

Further analysis examined the moderating roles of perceived social support (PSS) and self-esteem (SE) in shaping the association between help-seeking stigma (HSS) and PD. The interaction between HSS and PSS showed a negative association with PD (H2a: *β* = −0.087, *p* = 0.050), suggesting that stigma against help-seeking is particularly detrimental for students who also perceive limited social support. Similar findings have been reported among U.S. and European students, where stigma interacts with weak networks to suppress help-seeking (Eisenberger et al., 2020), though in China and other East Asian contexts stigma appears more deeply entrenched, reducing even informal coping (Hu et al., 2024). Conversely, the interaction between HSS and SE exhibited a positive association with PD (H2b: *β* = 0.099, *p* = 0.027). This finding indicates that high self-esteem may buffer the negative impact of stigma, echoing studies in Western universities where self-worth moderates distress outcomes (Topkaya, 2021; Yu et al., 2022).

The mediation analysis highlighted the pivotal role of psychological resources in shaping detachment. PSS partially mediated the association between AP and PD (H3a: *β* = −0.051, *p* = 0.077), suggesting that social support may attenuate the negative influence of perfectionistic concerns. However, PSS did not significantly mediate the DB–PD relationship (H3b: *β* = −0.020, *p* = 0.131), implying that social support alone may not counteract digital fatigue. This aligns with findings from European studies where behavioral regulation and digital literacy, rather than social support, drive recovery from digital overload (Kühnel et al., 2020).

Resilience (REL) emerged as a consistent and significant mediator across all pathways tested (H4a–H4d, all *p* < 0.05). This underscores resilience as a central mechanism enabling students to maintain detachment despite academic and cultural stressors. Cross-national research echoes this finding: U.S. and Australian studies confirm resilience as a buffer against academic burnout (Chen et al., 2022), while Korean and Japanese studies emphasize its role in mitigating stigma-related stress (Lee and Kim, 2023). Thus, while resilience is universally protective, the triggers and contexts of its activation differ.

Self-esteem also played an important mediating role, significantly mediating the associations between AP and FPS with PD (H5a: *β* = 0.146, *p* < 0.001; H5b: *β* = 0.068, *p* = 0.014). These results suggest that students with higher self-esteem are better able to manage both perfectionism and familial stress while sustaining psychological detachment. Similar protective effects have been observed in Western contexts where self-esteem underpins adaptive coping and detachment (Orth and Robins, 2022), though in collectivist societies self-esteem often interacts with family-based expectations, shaping both its availability and its efficacy.

Taken together, the results suggest that among the stressors examined, SCA exerts the strongest and most consistent association with reduced detachment. AP and DB influence detachment primarily through indirect mechanisms, while FPS plays a modest role shaped by cultural context. Across stressors, resilience and SE consistently function as psychological buffers, underscoring their universal protective role. PSS, meanwhile, demonstrates both mediating and moderating functions, highlighting the importance of external networks alongside internal resources. From an international perspective, these findings align with global research on student stress and detachment while also illustrating how cultural obligations (e.g., filial piety) and entrenched stigma amplify challenges unique to Chinese higher education.

## 5 Implications of this study

### 5.1 Theoretical implications

This study offers several key theoretical contributions by extending and integrating the Stressor-Strain-Outcome (SSO) Model, Conservation of Resources (COR) Theory, and Cognitive Appraisal Theory (CAT) within the context of psychological detachment in Chinese higher education. While these frameworks have been predominantly applied to occupational stress, this research demonstrates their conceptual relevance—and limitations—when applied to student populations embedded in collectivist, digitally saturated, and performance-oriented academic environments.

First, the findings enrich the SSO Model (Sonnentag and Kühnel, 2016) by illustrating how academic stressors—particularly social comparison anxiety, digital burnout, and filial piety stress—act as chronic antecedents of psychological strain that hinder students’ ability to detach from academic concerns. Unlike occupational contexts where work-rest boundaries are often externally defined, student environments are less structured and more permeable, with stress perpetuated by self-imposed perfectionism and continuous exposure to competitive digital platforms (Mäntymäki et al., 2022). This calls for a reconceptualization of “strain” in educational contexts to include not only emotional exhaustion but also culturally internalized moral pressures that inhibit recovery.

Second, the study contributes to COR Theory (Hobfoll, 2010) by reaffirming the role of psychological resources—resilience, self-esteem, and perceived social support—in buffering the negative effects of academic stress. However, it refines the theory by identifying help-seeking stigma as a sociocultural moderator that can erode or block access to these resources. This finding highlights a previously underexplored asymmetry in COR Theory: the mere possession of resources is not sufficient—students must also be willing and socially permitted to activate them. In contexts where educational success is equated with filial honor, the fear of appearing weak or burdensome deters students from utilizing available support, thus exacerbating resource depletion (Villatoro et al., 2022).

Third, by applying Cognitive Appraisal Theory (Lazarus and Folkman, 1984), the study shows that students’ interpretations of stress are filtered through sociocultural scripts. Students with higher self-esteem and perceived social support tend to appraise academic demands as challenges rather than threats, facilitating detachment. Conversely, stigma alters secondary appraisal by making coping resources seem socially inaccessible or illegitimate, especially in collectivist cultures that valorize endurance and emotional restraint (Poudel et al., 2020; Yu et al., 2022). This suggests that appraisal processes are not only psychological but culturally mediated, requiring an expansion of CAT to account for normative constraints on coping expression.

Finally, the inclusion of filial piety stress as a culturally grounded construct extends all three frameworks by demonstrating how cultural expectations act both as stressors and barriers to recovery. It challenges the Western-centric assumptions of agency and recovery embedded in these models and underscores the need for culturally nuanced stress frameworks that account for internalized obligations, digital overstimulation, and the moralization of academic engagement.

Collectively, this study advances theoretical discourse by showing that psychological detachment in high-performance academic cultures cannot be fully understood without integrating digital exposure, cultural norms, and sociocognitive barriers. It calls for future models of stress and recovery to move beyond universalist assumptions and adopt contextually adaptive frameworks that reflect the lived realities of student populations in non-Western settings.

### 5.2 Practical implications

The findings of this study carry direct implications for educators, institutions, policymakers, and mental health professionals seeking to strengthen student wellbeing and academic performance. The evidence indicates that academic perfectionism, social comparison anxiety, digital burnout, and filial piety stress can all undermine students’ ability to detach from academic concerns, underscoring the need for institutional responses that address both structural and individual barriers to recovery. Importantly, detachment cannot be reduced to an individual responsibility; it is shaped by academic culture, digital learning design, and sociocultural expectations.

A first set of implications concerns institutional learning environments. Universities can adopt structural reforms that temper perfectionistic pressures by redesigning assessment and grading schemes. For instance, policies such as *flexible grading bands, formative rather than exclusively summative assessment, and explicit recognition of effort and improvement* can reduce the anxiety associated with absolute performance standards. Introducing *flexible class timetables and designated low-intensity academic weeks* can also help reduce academic overload and allow for restorative breaks. Similarly, course design can incorporate deliberate pauses or “detachment moments” (e.g., short reflection breaks in lectures, non-assessed learning activities) to normalize disengagement as part of effective learning.

Second, the mediating role of resilience, self-esteem, and perceived social support highlights the value of targeted student development programs. Universities should invest in resilience-building workshops focusing on adaptive coping strategies, mindfulness training, and cognitive reappraisal techniques. *Peer mentoring systems*—pairing senior students with incoming students—can build support networks that reduce social comparison anxiety and encourage help-seeking. In parallel, *on-campus counseling services should be visible, affordable, and integrated with academic advising*, so that support is not perceived as a separate or stigmatized domain. For example, embedding counselors within faculties can encourage routine, low-barrier consultations.

Third, addressing digital burnout requires both behavioral and policy interventions. Universities could institutionalize “digital detox” practices by setting aside technology-free study zones in libraries or residence halls and encouraging device-free classroom discussions in selected courses. Digital literacy workshops should emphasize strategies such as scheduled screen breaks, use of productivity applications that monitor screen time, and alternating between online and offline study methods. Public health campaigns within the university—delivered via posters, short videos, or student-led initiatives—could highlight the benefits of balanced technology use and encourage healthy digital boundaries.

Fourth, given the salience of filial piety stress in this context, parent engagement must become part of institutional mental health strategy. Universities can design parent outreach programs that explain the effects of excessive pressure on student mental health, while also providing guidance on constructive ways to express support. Parent-student workshops at orientation or mid-semester checkpoints could help align familial expectations with the student’s actual academic journey. Faculty advisors, in turn, should be trained to recognize signs of parental over-involvement and to counsel students on negotiating academic obligations with respect for cultural values but without compromising wellbeing.

Finally, the moderating role of help-seeking stigma highlights the need for interventions that do more than make resources available—they must also reshape cultural norms. Universities should launch awareness campaigns featuring student testimonials and peer champions to normalize counseling and reduce stigma. Faculty and teaching staff can be equipped with *basic mental health literacy* so that wellbeing becomes part of everyday academic discourse rather than a specialist issue. Policy-level initiatives might include *mandatory wellbeing check-ins* during critical academic periods (e.g., exam seasons) or structured recovery breaks for students reporting high levels of strain. Such measures signal an institutional commitment to detachment as a legitimate and necessary component of academic success.

In sum, universities should adopt a multi-layered approach: redesigning academic structures to reduce perfectionism-driven strain, building resilience and self-esteem through targeted programs, regulating digital engagement, involving families in mental health conversations, and actively dismantling stigma around help-seeking. These concrete steps can help transform high-pressure academic environments into ecosystems that balance achievement with sustainability, ensuring that students not only perform but also preserve their psychological health in the long term.

## 6 Conclusion and future research

This study applied the Stressor–Strain–Outcome (SSO) Model, Conservation of Resources (COR) Theory, and Cognitive Appraisal Theory to examine how academic perfectionism, social comparison anxiety, digital burnout, and filial piety stress hinder students’ ability to disengage from their studies. Findings suggest that social comparison anxiety and digital burnout exert particularly strong burdens, perpetuating rumination and limiting students’ capacity for recovery. At the same time, resilience, self-esteem, and perceived social support emerged as significant protective resources, while help-seeking stigma revealed the cultural constraints that reduce the effective use of these resources. Together, these results extend stress-adaptation frameworks by integrating cultural and digital dimensions into the study of psychological detachment.

Although culturally situated in China, these findings extend globally relevant stress-coping frameworks by illustrating how cultural scripts (e.g., filial piety, stigma) intensify barriers to detachment. Cross-national research can adapt this model to both collectivist and individualist settings, refining understanding of how universal stressors interact with cultural norms to shape psychological recovery.

### 6.1 Limitations

Several limitations warrant critical reflection. First, the reliance on self-reported data introduces the risk of common method variance and social desirability bias, despite the use of a time-lagged design. Incorporating physiological indicators (e.g., cortisol levels, digital usage logs) or behavioral measures in future research could improve validity. Second, although purposive sampling in Zhejiang Province ensured cultural relevance, it limits generalizability to other regions of China or to international contexts where academic and familial expectations differ. Future work should replicate this model in Western and non-Asian collectivist contexts to evaluate whether cultural norms or digital practices drive the strongest barriers to detachment. Third, while the theoretical framing combined SSO, COR, and Cognitive Appraisal Theory, the cross-sectional mediation and moderation tests cannot fully capture dynamic feedback loops. Future research should therefore apply longitudinal or diary-based designs to track how resource depletion and replenishment unfold over time. Finally, the study focused on higher education students; extending inquiry to younger populations (e.g., high school students preparing for university entrance exams) or working professionals may yield additional insights into how psychological detachment functions across life stages.

### 6.2 Future research directions

Building on these limitations, several pathways merit attention. Longitudinal and experience-sampling methods should be employed to capture within-person fluctuations in detachment and recovery, allowing researchers to test how coping strategies evolve across academic terms or high-stakes periods such as exam seasons. Cross-cultural investigations could examine how cultural values—such as filial piety in East Asia versus individual autonomy in Western contexts—shape students’ willingness and ability to detach, offering a richer understanding of the interplay between cultural norms and psychological resources. In particular, comparative research across China, South Korea, Japan, and Western universities could clarify whether cultural expectations or technological environments exert the stronger influence on detachment. Intervention-based studies are particularly critical: resilience training modules, parent-student workshops, stigma-reduction campaigns, and structured digital detox initiatives could be experimentally tested to determine their efficacy in improving detachment and academic wellbeing. Digital tools such as AI-driven tutoring systems, mindfulness applications, or gamified stress management platforms should also be evaluated as scalable supports against academic rumination and digital burnout. At the systemic level, policy-focused research could investigate how flexible workload arrangements, alternative grading systems (e.g., pass/fail or narrative assessment), and embedded counseling services impact students’ ability to sustain healthy recovery cycles.

By critically engaging with methodological constraints, contextual boundaries, and theoretical gaps, this study paves the way for more robust inquiry into psychological detachment in higher education. Future research that integrates cross-cultural comparisons, longitudinal assessments, and intervention trials will not only refine theoretical models but also provide actionable guidance for universities striving to foster environments that balance academic achievement with psychological resilience.

## Data Availability

The raw data supporting the conclusions of this article will be made available by the authors, without undue reservation.
